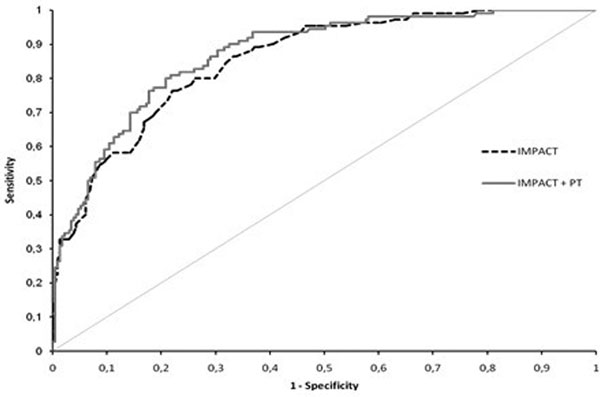# Predictive ability of the IMPACT-TBI prognostic model is improved by adding markers of coagulation

**DOI:** 10.1186/cc12260

**Published:** 2013-03-19

**Authors:** R Raj, J Siironen, R Kivisaari, L Handolin, J Hernesniemi, M Skrifvars

**Affiliations:** 1 Helsinki University Central Hospital, Helsinki, Finland

## Introduction

One of the most used prognostic models for traumatic brain injury is the IMPACT-TBI model, which predicts 6-month mortality and unfavorable outcome. Our aim was to study whether adding markers of coagulation improves the model's predictive power when accounting for extracranial injury.

## Methods

Patients with a TBI admitted to a designated trauma center in 2009/10 were screened retrospectively and included according to the IMPACT study criteria. The predictive outcome was calculated for included patients using the full IMPACT-TBI model. To assess coagulopathy and extracranial injury we used the prothrombin time percentage (PT), platelet count (10^9^), and injury severity score (ISS). PT, platelets and ISS were added to the IMPACT model, creating new models. The predictive power of the new models was tested by comparing their area under the receiver operating characteristic curve (AUC) with the original model.

## Results

Totally 342 were included. Six-month mortality was 32% and unfavorable outcome 57%. The IMPACT model's predictive power for mortality and neurological outcome; AUC: 0.85, CI: 0.81 to 0.89; and AUC: 0.81, CI: 0.76 to 0.86. Nonsurvivors and patients with an unfavorable outcome had lower platelets (159; 165 vs. 191; 198), PT (76; 77 vs. 86; 90) and higher ISS (50; 44% ISS >25 vs. 34; 32% ISS >25) compared with survivors and favorable outcome (*P <*0.05). Addition of PT but not ISS nor platelets resulted in a significant improvement of the IMPACT model's predictive power for 6-month mortality; AUC: 0.87, CI: 0.81 to 0.89, *P *= 0.017 (Figure 1). In multivariate analysis PT remained an independent significant predictor of outcome when adjusting for IMPACT prognosis and ISS (OR: 0.985, CI: 0.975 to 0.996).

## Conclusion

Addition of PT to the IMPACT model improves the predictive power for 6-month mortality but not neurological outcome when adjusting for ISS.

**Figure 1:**